# Implementation of a customised antimicrobial resistance laboratory scorecard in Cameroon, Ethiopia and Kenya

**DOI:** 10.4102/ajlm.v11i1.1476

**Published:** 2022-06-20

**Authors:** André Trollip, Renuka Gadde, Tjeerd Datema, Kamau Gatwechi, Linda Oskam, Zachary Katz, Andrew Whitelaw, Peter Kinyanjui, Patrick Njukeng, Dawit A. Wendifraw, Ibrahimm Mugerwa, Grace Najjuka, Nicholas Dayie, Japheth A. Opintan, Heidi Albert

**Affiliations:** 1 Foundation for Innovative New Diagnostics (FIND) South Africa, Cape Town, South Africa; 2 Becton, Dickinson & Company, Franklin Lakes, New Jersey, United States; 3 DATOS, Leiden, the Netherlands; 4 Becton, Dickinson & Company, Nairobi, Kenya; 5 Foundation for Innovative New Diagnostics (FIND), Geneva, Switzerland; 6 Department of Pathology, Faculty of Medicine and Health Sciences, Stellenbosch University, South Africa; 7 National Health Laboratory Service, Tygerberg Hospital, Cape Town, South Africa; 8 National Public Health Laboratory, Kenyatta National Hospital, Nairobi, Kenya; 9 Global Health Systems Solutions, Isokolo, Cameroon; 10 National Clinical Bacteriology and Mycology Reference Laboratory, Ethiopian Public Health Institute, Addis Ababa, Ethiopia; 11 Ministry of Health, National Health Laboratories and Diagnostic Services-AMR-National Coordination Centre, Kampala, Uganda; 12 National Health Laboratories and Diagnostic Services, Kampala, Uganda; 13 Department of Medical Microbiology, University of Ghana Medical School, Accra, Ghana

**Keywords:** antimicrobial resistance, laboratory, clinical, blood, urine, faeces

## Abstract

**Background:**

In low-resource settings, antimicrobial resistance (AMR) is detected by traditional culture-based methods and ensuring the quality of such services is a challenge. The AMR Scorecard provides laboratories with a technical assessment tool for strengthening the quality of bacterial culture, identification, and antimicrobial testing procedures.

**Objective:**

To evaluate the performance of the AMR Scorecard in 11 pilot laboratory evaluations in three countries also assessed with the Stepwise Laboratory Quality Improvement Process Towards Accreditation (SLIPTA) checklist.

**Methods:**

Pilot laboratory evaluations were conducted in Cameroon, Ethiopia and Kenya between February 2019 and March 2019. Assessors with previous SLIPTA and microbiology experience were trained. Assessors performed the laboratory assessments using the SLIPTA and AMR Scorecard tools.

**Results:**

Weaknesses in technical procedures and the quality management systems were identified in all areas and all laboratories. Safety had the highest mean performance score (SLIPTA: 68%; AMR Scorecard: 73%) while management review had the lowest (SLIPTA: 32%; AMR Scorecard: 8%) across all laboratories. The AMR Scorecard scores were generally consistent with SLIPTA scores. The AMR Scorecard identified technical weaknesses in AMR testing, and SLIPTA identified weaknesses in the quality management systems in the laboratories.

**Conclusion:**

Since the AMR Scorecard identified important gaps in AMR testing not detected by SLIPTA, it is recommended that microbiology laboratories use SLIPTA and the AMR Scorecard in parallel when preparing for accreditation. Expanding the use of the AMR Scorecard is a priority to address the need for quality clinical microbiology laboratory services in support of optimal patient care and AMR surveillance.

## Introduction

Antimicrobial resistance (AMR) is a global problem, with resistant infections currently claiming at least 50 000 lives each year across Europe and the United States alone and hundreds of thousands more in other areas of the world.^1^ Knowledge of AMR patterns is essential for optimal individual patient care, antimicrobial stewardship and AMR surveillance.^2^ Reviews of available data from Africa have found a high level of resistance to commonly used antibiotics in the region.^3,4^ Despite nine new African countries joining the World Health Organization’s Global Antimicrobial Resistance Surveillance System in 2020/2021, AMR data are generally lacking in many low- and middle-income countries (LMICs).^5^ In addition to this limited availability, there are also concerns over the quality of existing AMR data.^6^ During the current coronavirus disease 2019 pandemic, antimicrobial stewardship activities have been impacted globally, requiring coordinated strategies to inform actions to reduce the potential longer-term impact on AMR.^7^

While recent advances in molecular methods to detect AMR are being increasingly implemented in high-income settings,^8,9^ these are not available in many LMICs, and traditional culture-based diagnostic methods, performed by clinical microbiology services, remain the gold standard. Ensuring the quality of such services is a challenge because, in addition to the pre-analytical, analytical and post-analytical phases that take place within the laboratory, there are numerous other key drivers of overall diagnostic quality,^10^ including clinical question formulation and test selection, test ordering, sample collection and transportation to the laboratory, testing and results reporting, test results interpretation, and patient follow-up for clinical management or referral for further testing. Laboratories with weak systems have higher levels of errors, which can affect patient care and undermine the confidence that clinicians have in laboratory services.^11,12^ To address the coronavirus disease 2019 pandemic, laboratories are receiving new molecular and point-of-care technologies, thus increasing the number of samples processed and reinforcing the need for high-performing and high-quality laboratories and systems, particularly in LMICs.

Significant advances have been made towards improving laboratory capacity and quality in disease areas such as HIV and tuberculosis. One example is the Stepwise Laboratory Quality Improvement Process Towards Accreditation (SLIPTA) initiative developed by the United States Centers for Disease Control and Prevention in collaboration with the American Society for Clinical Pathology, the Clinton Health Access Initiative, and the World Health Organization Regional Office for Africa to promote the uptake of quality improvement initiatives in LMIC laboratories.^13,14,15^ However, only a few clinical microbiology laboratories in LMICs have achieved any form of accreditation, that is, a formal recognition that their quality management system (QMS) complies with international standards.^16,17,18^

Although the SLIPTA initiative has, to some extent, facilitated laboratory improvement in Africa, it may not specifically address the quality of processes in AMR laboratories, including sample culture, species identification, and susceptibility testing. While implementing QMS elements is critical, improving compliance with a technical standard of testing is equally important.

In recognition of the potential gap in the quality of AMR-related testing, we developed the AMR Laboratory Scorecard (AMR Scorecard),^19^ which aims to improve the appropriate use of diagnostics to identify pathogens and guide patient treatment and management, and to optimise the surveillance and early detection of AMR. The AMR Scorecard focuses on priority specimen types such as blood, urine, and faecal samples, and includes the culture, pathogen detection, species identification, and antimicrobial susceptibility testing (AST) processes. The AMR Scorecard is designed to assess these technical processes for the priority pathogens reported to the Global Antimicrobial Resistance Surveillance System. This includes pathogens associated with hospital and community-acquired infections in which AMR is reportedly increasing, threatening the use of key drugs.^5^ In this article, we describe the performance of the developed AMR Scorecard during pilot laboratory evaluations in three countries.

## Methods

### Ethical considerations

This article describes the performance of the AMR Scorecard during pilot evaluations and does not require ethical clearance. This article followed all ethical standards for research without direct contact with human or animal subjects.

### Study design

Pilot evaluations of the AMR Scorecard were conducted in Cameroon, Ethiopia, and Kenya between February and March 2019. The countries were selected based on their enrolment in the Global Antimicrobial Resistance Surveillance System and engagement with Becton Dickinson or Foundation for Innovative New Diagnostics (FIND), the global alliance for diagnostics, in ongoing laboratory strengthening activities. The laboratories in these five countries are representative of microbiology services across the diagnostic network from central level laboratories to district-level laboratories. Assessors were selected and trained on the use of the AMR Scorecard before performing the pilot laboratory assessments.

### Antimicrobial resistance scorecard

The AMR Scorecard was developed based on the latest guidance and requirements for AMR testing obtained from a review of existing tools, checklists, and guidelines, including those of the Healthcare-Associated Infection Surveillance India^20^ and the United States Centers for Disease Control and Prevention.^21^

The AMR Scorecard is based on the World Health Organization Regional Office for Africa SLIPTA Checklist version 2:2015 and incorporates clinical microbiology laboratory-specific requirements linked to sub-clauses in the SLIPTA checklist. It consists of three scorecard modules that are used to assess the technical procedures for processing blood, urine, and faecal samples. Assessment of technical procedures includes questions to determine if isolation procedures (e.g. ‘Are media used for primary culture of faeces incubated at 35 °C – 37 °C for at least 18 hours?’), identification procedures (e.g. ‘Are Gram stains performed for all blood cultures showing any sign of positive growth [e.g. turbidity, haemolysis, or gas production]?’) and AST procedures (e.g. ‘Does the laboratory use Combination Disk Test or another equivalent method for carbapenemase screening?’) are being performed according to microbiology best practices.

The AMR Scorecard is designed to be used as a stand-alone internal assessment tool or as part of a comprehensive SLIPTA assessment to ensure the application of SLIPTA requirements to these test methods. The AMR Scorecard uses the same scoring convention and the same 12-section structure as the SLIPTA checklist. Individual AMR testing modules are scored according to the percentage of requirements met in each modular checklist. However, unlike SLIPTA, whose laboratory assessment is based on the International Standards Organization (ISO) 15 189^22^ standard, the AMR Scorecard assessment compares technical laboratory practices against best practices for microbiology and AMR.

An AMR Scorecard eTool was developed to supplement the hard-copy technical modules and SLIPTA checklist and to allow automated analyses and reporting of the laboratory assessments.^22^ The eTool consists of a general AMR testing spreadsheet for recording responses to questions common to all the technical modules, spreadsheets for information specific to faeces, urine and blood, a spreadsheet for previous audit information, and a summary spreadsheet with automated analysis and visualisation of the technical assessment scores. A spreadsheet corresponding to the 12 sections of SLIPTA is also provided to allow simultaneous SLIPTA evaluations. The summary spreadsheet with automated analysis provides a detailed overview and visualisation of the SLIPTA assessment scores.

### Assessor training

Trainee assessors who had microbiology experience and had participated in SLIPTA assessments in their laboratories (but were not necessarily African Society for Laboratory Medicine SLIPTA certified) were chosen to attend the training workshops. Training on the AMR Scorecard was conducted in Ethiopia from 11 to 13 February 2019, in Kenya from 25 to 26 February 2019, in Uganda on 18 February 2020, and in Ghana on 26 February 2020. Facilitators trained two assessors from Cameroon, eight assessors from Ethiopia, and six assessors from Kenya on the interpretation of AMR Scorecard questions, the use of the eTool, and the procedures for conducting the assessments. As all the trainee assessors were already familiar with the use of the SLIPTA checklist, training on SLIPTA was not provided.

Theoretical training was supplemented by practical assessments of three facilities (two in Ethiopia and one in Kenya). The national or reference laboratories were chosen for the practical assessment to provide trainee assessors exposure to all the procedures evaluated using the scorecard (Table 1). All three laboratories performed basic urine and faeces culture. Automated blood cultures were performed by two laboratories, one in Ethiopia and one in Kenya. The reference laboratories in Ethiopia and Kenya are ISO 15189:2012 certified and perform automated identification and AST. The practical assessments were overseen by the facilitators. Due to time constraints, SLIPTA assessments were not performed during the training assessments.

**TABLE 1:** Antimicrobial resistance laboratory scorecard assessment activities in 14 laboratories in Cameroon, Ethiopia, and Kenya between February 2019 and March 2019.

Laboratory	Level	Assessment type	Date of assessment	Assessment partners	Microbiological methods used in the laboratory
**Cameroon**					
A	District	SLIPTA and AMR Scorecard	18 February 2019 and 1 March 2019	GHSS	Manual cultures. Conventional ID and AST
B	Regional reference				Basic culture: urine, faeces and manual blood cultures. Automated or kit-based and conventional ID and AST
C	Provincial				Basic culture: urine, faeces and manual blood cultures. Kit-based and conventional ID and AST
**Ethiopia**					
D	Regional reference	SLIPTA and AMR Scorecard	14 and 20 February 2019	EPHI and FIND	Manual cultures. Conventional ID and AST
E	District				Manual cultures. Conventional ID and AST
F	Private				Manual cultures. Conventional ID and AST
G	National reference	Training assessment, AMR Scorecard only			Basic culture: urine and faeces. Automated blood cultures. Automated or kit-based and conventional ID and AST. In-house molecular methods available for research only
H	Regional reference	SLIPTA and AMR Scorecard			Manual cultures. Conventional ID and AST
I	District	Training assessment, AMR Scorecard only			Manual cultures. Conventional ID and AST
J	Zonal	SLIPTA and AMR Scorecard			Manual cultures. Conventional ID and AST
K	District				Manual cultures. Conventional ID and AST
**Kenya**					
L	Provincial	SLIPTA and AMR Scorecard	26 February 2019 and 1 March 2019	BD and MoH, Kenya	Basic culture: urine, faeces and manual blood cultures. Automated or kit-based and conventional ID and AST
M	National	Training assessment, AMR Scorecard only			Basic culture: urine and faeces. Automated blood cultures. Conventional ID and AST
N	Provincial	SLIPTA and AMR Scorecard			Basic culture: urine and faeces. Automated blood cultures. Conventional ID and AST

FIND, Foundation for Innovative New Diagnostics; ID, identification; AST, antimicrobial susceptibility testing; SLIPTA, Stepwise Laboratory Quality Improvement Process Towards Accreditation; AMR, antimicrobial resistance; GHSS, Global Health Systems Solutions; EPHI, Ethiopian Public Health Institute; MoH, Ministry of Health; BD, Becton Dickinson.

### Pilot laboratory assessments

Before commencing the pilot assessments at the respective laboratories, assessors introduced the AMR Scorecard to the laboratory head and quality officer. All the assessments were conducted using the SLIPTA checklist and the AMR Scorecard and transferred to the eTool for further analysis.

Assessors evaluated the laboratory operations based on the SLIPTA checklist and AMR Scorecard items, recording scores for each item and documenting findings in detail. During the assessment, assessors reviewed laboratory documentation to verify that policies, manuals, and standard operating procedures were complete, current, and accurate. They also reviewed records and observed laboratory procedures to verify that AMR policies were being followed and that laboratory procedures used were appropriate for the testing performed. In addition, the assessors determined the availability of functional and well-maintained equipment, reviewed data on the number of processed samples, isolates, contaminated cultures, and negative cultures for each sample type, and reviewed the internal quality control and external quality assessment results. These data provided assessors with an overview of the laboratories’ operations and allowed the identification of systemic technical issues not easily determined using SLIPTA alone.

Following the assessments, the assessors provided feedback to the laboratory head, quality officer, and laboratory technologists. Non-conformities with the ISO 15189:2012 standard identified by SLIPTA^23^ and non-conformities with microbiology best practices identified by the AMR Scorecard were tabulated and presented to the laboratory along with copies of the completed checklists.

### Data management

The results of the pilot laboratory assessments were transferred to the eTool, which then automatically calculated the scores and totals for each section and generated a bar graph of laboratory performance by section. The AMR Scorecard results for blood, faeces, and urine were analysed with the SLIPTA scores.

## Results

### Antimicrobial resistance laboratory scorecard pilot assessments

**FIGURE 1:**
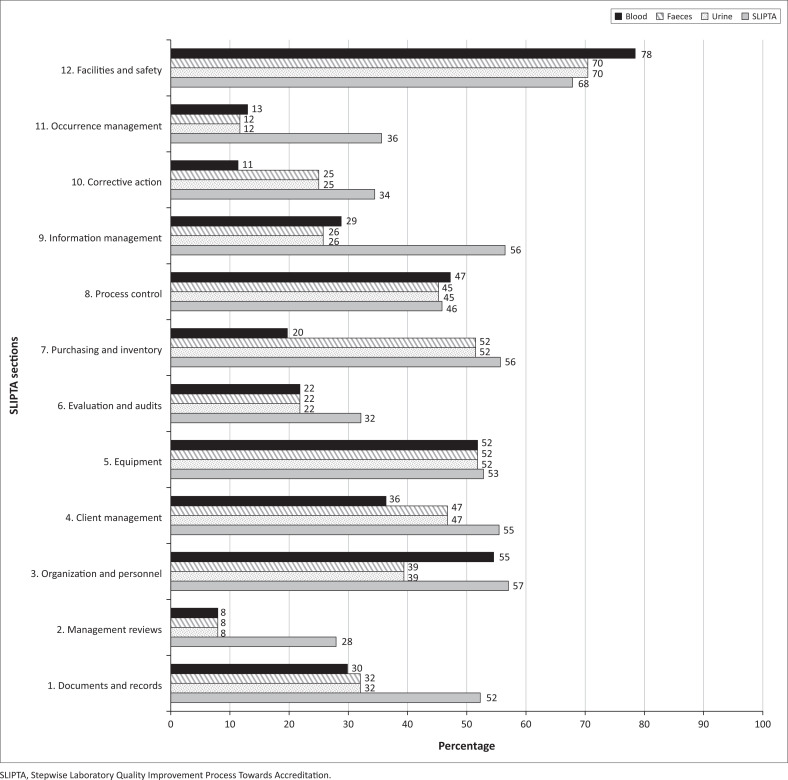
Mean performance scores of 11 microbiology laboratories assessed with the antimicrobial resistance laboratory scorecard and SLIPTA in Cameroon, Ethiopia, and Kenya between February 2019 and March 2019.

In the pilot assessments conducted in this study, weaknesses in technical procedures and the QMS were identified in all areas and all laboratories (Figure 1). The mean AMR Scorecard assessment scores ranged between 8% (Section 2: Management Reviews) and 73% (Section 12: Facilities and Safety), and the mean SLIPTA scores ranged between 32% (Section 2: Management Reviews) and 68% (Section 12: Facilities and Safety).

Based on the AMR Scorecard assessments, all 11 laboratories performed best with all sample types in Section 12: Facilities and Safety (range: 25% – 100%; mean: 73%). The weakest performance for all sample types in all laboratories was in Section 2: Management Reviews (range: 0% – 13%; mean: 8%), followed by Section 11: Occurrence Management (range: 0% – 71%; mean: 12%), and Section 6: Evaluations and Audits (range: 0% – 100%; mean: 22%).

Technical issues with the processing of all sample types (isolation, identification, and AST) were identified in Section 8: Process Control and Internal and External Quality Assessment of the AMR Scorecard (range: 3% – 72%; mean: 46%). In 10 of 11 laboratories, data on the number of isolated pathogens and cumulative AST patterns were not collected and reported to the relevant oversight committees such as the antimicrobial stewardship committee, or hospital surveillance or outbreak team.

Technical issues with the processing of urine samples included failure to perform cell counts or wet preparations (6 of 11 laboratories), lack of rejection criteria (8 of 11 laboratories), lack of quality controls for media (4 of 11 laboratories), lack of antibiotic discs for AST (9 of 11 laboratories), and failure to use purity plates or standardised inocula for AST (7 of 11 laboratories).

Technical issues with the processing of faeces samples included failure to perform wet preparations for parasites (5 of 11 laboratories), shortage of Selenite F Broth and lack of sub-culture testing (8 of 11 laboratories), and failure to perform serological identification of either *Salmonella* or *Shigella* species (5 of 11 laboratories).

Technical issues with the processing of blood samples included failure to perform extended-spectrum beta-lactamase and carbapenemase detection tests (11 of 11 laboratories).

As with the results of the AMR Scorecard assessments, the SLIPTA assessment also identified Section 12: Facilities and Safety to be the strongest area (range: 40% – 95%; mean: 68%) in all the laboratories, followed by Section 3: Organization and Personnel (range: 9% – 91%; mean: 57%) and Section 7: Purchasing and Inventory (range: 0% – 88%; mean: 56%). The weakest area was Section 2: Management Reviews (range: 0% – 43%; mean: 28%). Some reasons for low SLIPTA scores included failure to conduct regular management reviews or audits (6 of 11 laboratories), and failure to collect and analyse quality indicators (6 of 11 laboratories). Weaknesses specific to the QMS were identified using SLIPTA as these are not assessed by the AMR Scorecard. For example, the content of the quality manual (Documents and Records) was identified as a weakness in 7 of 11 laboratories.

### Comparisons between the AMR scorecard and SLIPTA scores

The assessment scores obtained using the AMR Scorecard and SLIPTA were disaggregated by laboratory area and laboratory level (Tables 2–4). Two laboratories were designated as central, five as regional and four as district laboratories. The central level laboratories had a mean AMR Scorecard assessment score of 37% and a mean SLIPTA score of 45%. The regional-level laboratories had a mean AMR Scorecard assessment score of 40% and a mean SLIPTA score of 55%. The district-level laboratories had a mean AMR Scorecard assessment score of 29% and a mean SLIPTA score of 39%.

**TABLE 2:** Stepwise Laboratory Quality Improvement Process Towards Accreditation and AMR Laboratory Scorecard mean assessment scores in two central microbiology laboratories (A and B) in Cameroon and Ethiopia between February 2019 and March 2019.

Sections	A (%)				B (%)			
	SLIPTA	Urine	Faeces	Blood	SLIPTA	Urine	Faeces	Blood
1. Documents and records	29	17	11	11	61	47	47	53
2. Management reviews	7	0	0	0	43	0	0	0
3. Organization and personnel	27	0	17	17	64	33	33	100
4. Client management	50	86	86	57	80	86	86	57
5. Equipment	29	80	80	80	70	40	40	40
6. Evaluation and audits	13	0	0	0	7	20	20	20
7. Purchasing and inventory	38	33	33	17	67	67	67	33
8. Process control	37	41	47	52	63	53	52	63
9. Information management	52	33	33	17	74	50	67	50
10. Corrective action	32	0	0	0	47	25	25	13
11. Occurrence management	42	0	0	0	8	14	14	14
12. Facilities and safety	60	75	75	88	86	75	75	88

SLIPTA, Stepwise Laboratory Quality Improvement Process Towards Accreditation.

**TABLE 3:** Stepwise Laboratory Quality Improvement Process Towards Accreditation and AMR Laboratory Scorecard mean assessment scores in five regional microbiology laboratories (A–E) in Cameroon, Ethiopia, and Kenya between February 2019 and March 2019.

Sections	A (%)				B (%)				C (%)				D (%)				E (%)			
	SLIPTA	Urine	Faeces	Blood	SLIPTA	Urine	Faeces	Blood	SLIPTA	Urine	Faeces	Blood	SLIPTA	Urine	Faeces	Blood	SLIPTA	Urine	Faeces	Blood
1. Documents and records	7	0	8	0	75	75	75	75	64	28	28	28	82	33	33	28	68	33	20	33
2. Management reviews	0	0	0	0	57	13	13	13	36	13	13	13	43	13	13	13	43	13	13	13
3. Organization and personnel	9	0	0	0	73	100	100	100	82	67	67	67	91	100	100	100	73	0	67	67
4. Client management	0	0	0	0	90	57	57	71	70	57	57	57	90	86	86	86	70	71	43	14
5. Equipment	3	0	0	0	85	60	60	60	74	60	60	60	83	100	100	100	54	50	50	50
6. Evaluation and audits	0	0	0	0	47	20	20	20	13	20	20	20	100	100	100	100	73	20	20	20
7. Purchasing and inventory	0	0	0	0	75	67	67	33	88	100	100	33	83	100	100	33	71	100	100	33
8. Process control	9	3	8	5	60	68	63	72	41	67	72	56	67	67	69	70	56	44	41	43
9. Information management	21	0	33	0	87	50	50	67	53	0	0	0	76	33	67	33	63	0	0	0
10. Corrective action	11	25	25	0	58	100	100	13	32	25	25	13	79	25	25	13	37	25	25	0
11. Occurrence management	0	0	0	0	42	14	14	14	83	0	14	14	50	14	0	14	75	0	0	0
12. Facilities and safety	40	25	25	63	81	100	100	100	88	100	100	100	95	75	75	88	53	50	50	50

SLIPTA, Stepwise Laboratory Quality Improvement Process Towards Accreditation.

**TABLE 4:** Stepwise Laboratory Quality Improvement Process Towards Accreditation and AMR Laboratory Scorecard mean assessment scores in four district microbiology laboratories (A–D) in Cameroon, Ethiopia, and Kenya between February 2019 and March 2019.

Sections	A (%)				B (%)				C (%)				D (%)			
	SLIPTA	Urine	Faeces	Blood	SLIPTA	Urine	Faeces	Blood	SLIPTA	Urine	Faeces	Blood	SLIPTA	Urine	Faeces	Blood
1. Documents and records	11	11	6	0	68	33	33	33	61	42	33	42	50	33	25	25
2. Management reviews	0	0	0	0	43	13	13	13	7	13	13	13	29	13	13	13
3. Organization and personnel	18	17	0	0	73	67	67	67	50	33	17	67	68	17	33	17
4. Client management	0	14	14	0	60	43	43	43	30	14	14	14	70	0	0	0
5. Equipment	23	60	60	60	70	80	80	80	36	30	30	30	55	10	10	10
6. Evaluation and audits	0	0	0	0	47	20	20	20	13	20	20	20	40	20	20	20
7. Purchasing and inventory	33	0	0	0	75	67	50	33	29	17	17	0	54	17	17	0
8. Process control	47	38	50	10	57	56	53	72	34	33	37	60	33	29	25	16
9. Information management	32	0	17	0	100	17	0	17	32	67	100	100	32	33	33	33
10. Corrective action	0	0	0	0	53	25	25	13	21	25	25	63	11	0	0	0
11. Occurrence management	0	0	0	0	17	14	14	14	25	71	57	71	50	0	0	0
12. Facilities and safety	44	75	75	38	86	100	100	100	51	75	75	88	60	25	25	63

SLIPTA, Stepwise Laboratory Quality Improvement Process Towards Accreditation.

At least one laboratory performed poorly at each level. At the central level, Laboratory A had the lowest performance, with a mean AMR Scorecard assessment score of 30% and a mean SLIPTA score of 35%. At the regional-level, Laboratory A had a mean AMR Scorecard assessment score of 6% and a mean SLIPTA score of 8%. At the district-level, Laboratory A had a mean AMR Scorecard assessment score of 15% and a mean SLIPTA score of 17%. As these results suggest, the overall AMR Scorecard scores were similar to the overall SLIPTA scores, irrespective of the laboratory performance. However, differences between the SLIPTA and AMR Scorecard assessment scores in the different areas of the laboratory were noted at all laboratory levels. At the central laboratories, the largest differences between the AMR Scorecard and SLIPTA scores occurred in Section 2: Management Reviews (0% vs 25%), Section 9: Information Management (42% vs 63%) and Section 10: Corrective Action (10% vs 39%). At the regional laboratories, the largest differences between the AMR Scorecard and SLIPTA scores occurred in Section 1: Documents and Records (33% vs 59%), Section 2: Management Reviews (10% vs 36%), Section 9: Information Management (22% vs 60%), and Section 11: Occurrence Management (7% vs 50%). Finally, at the district laboratories, the largest differences between the AMR Scorecard and SLIPTA scores occurred in Section 1: Documents and Records (26% vs 47%), Section 4: Client Management (17% vs 40%), and Section 7: Purchasing and Inventory (18% vs 48%). Where there were large differences (> 20%) in the scores, the SLIPTA score was always higher.

## Discussion

Quality clinical microbiology services are an essential element of the AMR response that enable the appropriate use of antibiotics, improve AMR surveillance, and reduce the development of resistance.^24^ While implementing QMS elements in the microbiology laboratory is critical, improving compliance to a technical standard of testing is equally important. The AMR Scorecard provides laboratories with a specific technical assessment tool for strengthening the quality of culture, identification, and AST laboratory procedures.

In the pilot assessments conducted in this study, the AMR Scorecard scores generally correlated well with the SLIPTA scores. Safety in the laboratory has been identified as an increasingly important subject because of the emergence of highly infectious diseases, including coronavirus disease 2019. Although safety has traditionally been regarded as a low-priority issue in developing countries,^25^ in both the AMR Scorecard and SLIPTA assessments, Section 12: Facilities and Safety was found to be the strongest area in the laboratories. This focus on safety, even in laboratories with weak systems (e.g. regional laboratory A), is encouraging.

The SLIPTA assessments identified weaknesses in the QMS of the microbiology laboratories assessed. In 6 of the 11 laboratories, weaknesses identified included failure to conduct regular management reviews or audits. These findings are consistent with previous reports that some of the weakest areas in the laboratory are internal auditing and the collection of quality indicator data.^11,15,16,26^ Overall, six laboratories (55%) received zero stars (< 55%) using the official SLIPTA system, with only two laboratories (18%) scoring two stars (between 65% and 74%; regional laboratory B) and three stars (between 75% and 84%; regional laboratory D). The SLIPTA scores in this study are consistent with the SLIPTA scores from 47 countries worldwide, including 23 countries in Africa, assessed using the Strengthening Laboratory Management Toward Accreditation methodology prior to the initiation of laboratory strengthening activities (i.e. at baseline). Yao et al.^16^ found that the mean score in these laboratories at baseline was 39% (median 37%), with 84% of the laboratories scoring zero stars (i.e. score < 55%). It has been suggested that microbiology laboratories are trailing other clinical laboratories in achieving accreditation.^27^

The overall poor performance recorded at each laboratory level suggests that providing quality microbiology services is a challenge across the tiered network. The exclusion of national or reference microbiology laboratories in the assessments likely resulted in the lower overall scores observed in the pilot. While national or reference microbiology laboratories were assessed using the AMR Scorecard during the training, SLIPTA assessments were not conducted due to time constraints and thus the AMR Scorecard results were not included in this report. In settings with limited resources, strengthening technical testing and QMS (including accreditation) is often initiated at the national level, suggesting that overall scores may have been higher had they been included.

By providing a specific technical focus, the AMR Scorecard identified important gaps in AMR technical testing not detected by SLIPTA alone. The AMR Scorecard assesses the step-by-step procedures for sample processing, bacterial isolation and identification, and AST. Approximately 44% of the AMR Scorecard focuses on these procedures in contrast to SLIPTA which has a limited focus on technical procedures. Topics covered by SLIPTA are also covered by the AMR Scorecard but the latter focuses on the specific details related to AMR. For example, SLIPTA assesses whether standard operating procedures for laboratory functions and technical and managerial procedures are available, while the AMR Scorecard assesses whether the laboratory has, for example, documented procedures for microscopic examination and urine cell count. As the AMR Scorecard identified important gaps in AMR testing not detected by SLIPTA alone, and SLIPTA identified specific weaknesses in the QMS that were beyond the scope of the AMR Scorecard, it is recommended that microbiology laboratories that require a comprehensive assessment or are developing their QMS through continuous improvement toward accreditation be assessed using both the SLIPTA and the AMR Scorecard in parallel. Thus, the AMR Scorecard can be used as an entry point into the QMS journey, with laboratories choosing to apply for SLIPTA certification (or ISO certification) after reaching a satisfactory level (equivalent to 3–4 stars on SLIPTA). It should be noted that the official star recognition system provided by African Society for Laboratory Medicine can only be obtained through the SLIPTA certification provided by the African Society for Laboratory Medicine Secretariat.^28^

The importance of data collection and analysis is also highlighted in several questions in the AMR Scorecard (e.g. ‘Are the following performance indicators collected – Number and percentage of urine cultures with cell counts > 10^5^ cells/mL?’) and assessors are encouraged to assist laboratories to collect and analyse their data. The clinical laboratory is a major source of healthcare data that can be used to inform health system-wide actions meant to improve diagnostic test utilisation, service efficiency, and patient outcomes.^29^

In these evaluations, cumulative quality indicator data on isolated pathogens and AST were not collected by 10 of the 11 laboratories assessed. This was due to the lack of automated instruments or an electronic laboratory information system, meaning that staff were required to manually record and calculate isolation rates and AST patterns. In one laboratory, the compilation of data by the assessors exposed a very low isolation rate of enteric pathogens that required further investigation. Quality indicator data, if available, could have been used to identify the cause of the low isolation rate, thereby improving the quality of enteric bacterial culture.

In addition to improving the quality of laboratory testing, laboratory data can also be used to influence clinical decisions. For example, cumulative data on pathogens and AST results can be used to inform treatment guidelines. However, for laboratory data to impact health systems in such a way, the laboratory needs to carefully consider how the data are collated, communicated, and disseminated. Data from the assessments in this pilot revealed that 10 of the 11 laboratories failed to collect data on cumulative AST patterns and report these data to oversight committees, thereby missing the opportunity to inform antimicrobial stewardship decisions with laboratory data.

In the pilot assessments, where there were differences between the AMR Scorecard assessment and SLIPTA scores, the SLIPTA scores were consistently higher. In addition to the differences in the content of the two assessment tools, other factors may contribute to this finding. First, the laboratories included in the pilot were not part of any active and ongoing programmes to improve laboratory quality such as the Strengthening Laboratory Management Toward Accreditation programme.^15^ Only 2 of the 11 laboratories reported a previous SLIPTA assessment. It is expected that laboratories participating in quality improvement programmes are more likely to score higher on both assessment tools. Second, the AMR Scorecard is based on microbiology best practices and not on an ISO standard such as SLIPTA. Without a standard to guide preparations, it may not be clear to laboratories what requirements need to be in place to assure quality AMR testing. In this respect, the AMR Scorecard has a role to play in educating laboratories regarding the technical requirements for AMR testing.

There were several challenges to implementing the AMR Scorecard in the initial cohort of laboratories, including procurement of funding support for quality improvement and provision of cover for the trainers and mentors during programme-related absences. Mentoring of laboratories has been reported to be an important component of successful and sustainable quality improvement initiatives;^30^ thus, it is important to ensure that enough resources in terms of funding and personnel are put in place to allow mentoring to take place. Ideally, laboratories should be mentored by reference laboratories within the AMR surveillance network.

Based on feedback from facilitators and assessors, the AMR Scorecard needed to be revised to strengthen identified weaknesses. Suggested changes included revising the language of some questions and adding ‘Not Applicable’ options to others. Two additional questions to determine whether laboratories were performing extended-spectrum beta-lactamase and carbapenemase screening on faecal samples were added to the faeces module. This increased the score of the faeces module by four points. The total score of the blood and urine modules remained unaltered in the revised AMR Scorecard.

In 2020, the structure of the AMR Scorecard was changed, and additional scorecards were added to allow for assessments of other sample types including pulmonary, genital, and wound samples. Evaluations of the revised AMR Scorecard were performed in three laboratories in Ghana and Uganda.

### Limitations

The microbiology laboratories selected for the pilot of the AMR Scorecard do not represent the scope of microbiology technical abilities across Africa. While care was taken during the selection of laboratories for the study, the AMR Scorecard may be less useful in identifying and addressing gaps that impact the quality of testing in certain settings. In addition, all assessments, except those performed in Cameroon, included FIND and Becton Dickinson facilitators. As these facilitators were involved in the development of the AMR Scorecard, they may have influenced the outcomes of the assessments in favour of the AMR Scorecard.

### Conclusion

This study showed that a customised scorecard to guide the establishment and strengthening of AMR testing quality in resource-limited settings can assist in identifying and addressing quality gaps. The AMR Scorecard, used in conjunction with SLIPTA, found important gaps in the procedures for identification and AST of priority pathogens that were not identified by SLIPTA alone. Expanding the use of this scorecard will help address the need for quality clinical microbiology laboratory services to support optimal patient care and AMR surveillance.

## References

[1] O’Neill J. Review on antimicrobial resistance: Tackling a crisis for the health and wealth of nations [homepage on the Internet]. 2014 [cited 2020 Nov]. Available from: https://amr-review.org/sites/default/files/AMRReviewPaper-Tacklingacrisisforthehealthandwealthofnations_1.pdf

[2] Tangcharoensathien V, Chanvatika S, Sommanustweechaia A. Complex determinants of inappropriate use of antibiotics. Bull World Health Organ. 2018;96:141–144. 10.2471/BLT.17.199687 29403119 PMC5791781

[3] Ampaire L, Muhindo A, Orikiriza P, Mwanga-Amumpaire J, Bebell L, Boum Y. A review of antimicrobial resistance in East Africa. Afr J Lab Med. 2016;5(1):432. 10.4102/ajlm.v5i1.432 28879114 PMC5436405

[4] Leopold SJ, Van Leth F, Tarekegn H, Schultsz C. Antimicrobial resistance among clinically relevant bacterial isolates in sub-Saharan Africa: A systematic review. J Antimicrob Chemother. 2014;69(9):2337–2353. 10.1093/jac/dku176 24879668

[5] WHO. Global antimicrobial resistance and use surveillance system (GLASS) report 2021. Geneva: World Health Organization; 2021.

[6] Tadesse B, Ashley E, Ongarello S, et al. Antimicrobial resistance in Africa: A systematic review. BMC Infect Dis. 2017;17(1):616. 10.1186/s12879-017-2713-1 28893183 PMC5594539

[7] Rawson TM, Ming D, Ahmad R, et al. Antimicrobial use, drug-resistant infections and COVID-19. Nat Rev Microbiol. 2020;18:409–410. 10.1038/s41579-020-0395-y 32488173 PMC7264971

[8] Anjum M, Zankari E, Hasman H. Molecular methods for detection of antimicrobial resistance. Microbiol Spectr. 2017;5(6):1–17. 10.1128/microbiolspec.ARBA-0011-2017 PMC11687549 29219107

[9] Boolchandani M, D’Souza A, Dantas G. Sequencing-based methods and resources to study antimicrobial resistance. Nat Rev Genet. 2018;20(6):356–370. 10.1038/s41576-019-0108-4 PMC6525649 30886350

[10] Mesfin E, Taye B, Belay G, Ashenafi A, Girma V. Factors affecting quality of laboratory services in public and private health facilities in Addis Ababa, Ethiopia. eJIFCC. 2017;28(3):205–223. 29075171 PMC5655637

[11] Albert H, Trollip A, Erni D, et al. Developing a customised approach for strengthening tuberculosis laboratory quality management systems toward accreditation. Afr J Lab Med. 2017;6(2):576. 10.4102/ajlm.v6i2.576 28879165 PMC5523923

[12] Peter TF, Rotz PD, Blair DH, Khine A-A, Freeman RR, Murtagh MM. Impact of laboratory accreditation on patient care and the health system. Am J Clin Pathol. 2010; 134(4):550–555. 10.1309/AJCPH1SKQ1HNWGHF 20855635

[13] Alemnji GA, Zeh C, Yao K, Fonjungo PN. Strengthening national health laboratories in sub-Saharan Africa: A decade of remarkable progress. Trop Med Int Health. 2014;19(4):450–458. 10.1111/tmi.12269 24506521 PMC4826025

[14] Gershy-Damet GM, Rotz P, Cross D et al. The World Health Organization African region laboratory accreditation process: Improving the quality of laboratory systems in the African region. Am J Clin Pathol. 2010;134(3):393–400. 10.1309/AJCPTUUC2V1WJQBM 20716795

[15] Yao K, McKinney B, Murphy A, et al. Improving quality management systems of laboratories in developing countries: An innovative training approach to accelerate laboratory accreditation. Am J Clin Pathol. 2010;134(3):401–409. 10.1309/AJCPNBBL53FWUIQJ 20716796

[16] Yao K, Luman ET, SLMTA Collaborating Authors. Evidence from 617 laboratories in 47 countries for SLMTA-driven improvement in quality management systems. Afr J Lab Med. 2014;3(2):262. 10.4102/ajlm.v3i2.262 PMC4706175 26753132

[17] Kibet E, Moloo Z, Ojwang P, Sayed S, Mbuthia A, Adam R. Measurement of improvement achieved by participation in international laboratory accreditation in sub-Saharan Africa: The Aga Khan University Hospital Nairobi Experience. Am J Clin Pathol. 2014;141(2):188–195. 10.1309/AJCPV8A9MRWHGXEF 24436265

[18] Petti CA, Polage CR, Quinn TC, et al. Laboratory medicine in Africa: A barrier to effective health care. Clin Infect Dis. 2006;42(3):377–382. 10.1086/499363 16392084

[19] FIND. AMR laboratory scorecard package [homepage on the Internet]. [cited 2020 Nov]. Available from: https://www.finddx.org/amr-lab-scorecard-package/

[20] Healthcare Associated Infection Surveillance India. All India Institute of Medical Sciences (AIIMS), Centers for Disease Control and Prevention (CDC), Indian Council of Medical Research (ICMR). Laboratory antibacterial resistance surveillance readiness tool [homepage on the Internet]. 2017 [cited 2020 Nov]. Available from: https://www.haisindia.com/upload/fileuploads/1527836102_Lab.%20Assessment.pdf

[21] Centers for Disease Control and Prevention. Lab Assessment of Antibiotic Resistance Testing Capacity (LAARC) [homepage on the Internet]. 2021 [cited 2020 Nov]. Available from: https://www.cdc.gov/drugresistance/intl-activities/laarc.html

[22] ISO 15189. Medical laboratories – Requirements for quality and competence. Geneva: International Organization for Standardization; 2012.

[23] African Society for Laboratory Medicine. WHO AFRO SLIPTA checklist [homepage on the Internet]. 2007 [cited 2020 Nov]. Available from: http://www.afro.who.int/en/clusters-a-programmes/hss/blood-safety-laboratories-a-health-technology/blt-highlights/3859-who-guide-for-the-stepwise-laboratory-improvement-process-towards-accreditation-in-the-african-region-with-checklist.html

[24] Wilson ML. Assuring the quality of clinical microbiology test results. Clin Infect Dis. 2008;47(8):1077–1082. 10.1086/592071 18793099

[25] Ejilemele AA, Ojule AC. Health and safety in clinical laboratories in developing countries: Safety considerations. Niger J Med. 2004;13(2):182–188. 15293842

[26] Maina RN, Mengo DM, Mohamud AD, et al. Progressing beyond SLMTA: Are internal audits and corrective action the key drivers of quality improvement? Afr J Lab Med. 2014;3(2):222. 10.4102/ajlm.v3i2.222 29043193 PMC5637794

[27] Jacobs J, Hardy L, Semret M, et al. Diagnostic bacteriology in district hospitals in Sub-Saharan Africa: At the forefront of the containment of antimicrobial resistance. Front Med (Lausanne). 2019;6:205. 10.1086/592071 31608280 PMC6771306

[28] African Society for Laboratory Medicine. SLIPTA [homepage on the Internet]. 2021. Available from: https://aslm.org/what-we-do/#slipta

[29] Shirts B, Jackson B, Baird G, et al. Clinical laboratory analytics: Challenges and promise for an emerging discipline. J Pathol Inform. 2015;6:9. 10.4103/2153-3539.151919 25774320 PMC4355825

[30] Maruta T, Motebang D, Mathabo L, Rotz PJ, Wanyoike J, Peter T. Impact of mentorship on WHO-AFRO strengthening laboratory quality improvement process towards accreditation (SLIPTA). Afr J Lab Med. 2012;1(1):6. 10.4102/ajlm.v1i1.6 29062726 PMC5644515

